# KidneyGPS: a user-friendly web application to help prioritize kidney function genes and variants based on evidence from genome-wide association studies

**DOI:** 10.1186/s12859-023-05472-0

**Published:** 2023-09-21

**Authors:** Kira J. Stanzick, Klaus J. Stark, Mathias Gorski, Johannes Schödel, René Krüger, Florian Kronenberg, Richard Warth, Iris M. Heid, Thomas W. Winkler

**Affiliations:** 1 Department of Genetic Epidemiology, University of Regensburg, Regensburg, Germany; 2 Department of Nephrology and Hypertension, Friedrich-Alexander Universität Erlangen-Nürnberg, and Uniklinikum Erlangen, Erlangen, Germany; 3 Department of Genetics, Institute of Genetic Epidemiology, Medical University of Innsbruck, Innsbruck, Austria; 4 Medical Cell Biology, University of Regensburg, Regensburg, Germany

**Keywords:** GWAS, Kidney function, Web application, KidneyGPS, Gene prioritization

## Abstract

**Background:**

Genome-wide association studies (GWAS) have identified hundreds of genetic loci associated with kidney function. By combining these findings with post-GWAS information (e.g., statistical fine-mapping to identify independent association signals and to narrow down signals to causal variants; or different sources of annotation data), new hypotheses regarding physiology and disease aetiology can be obtained. These hypotheses need to be tested in laboratory experiments, for example, to identify new therapeutic targets. For this purpose, the evidence obtained from GWAS and post-GWAS analyses must be processed and presented in a way that they are easily accessible to kidney researchers without specific GWAS expertise.

**Main:**

Here we present KidneyGPS, a user-friendly web-application that combines genetic variant association for estimated glomerular filtration rate (eGFR) from the Chronic Kidney Disease Genetics consortium with annotation of (i) genetic variants with functional or regulatory effects (“SNP-to-gene” mapping), (ii) genes with kidney phenotypes in mice or human (“gene-to-phenotype”), and (iii) drugability of genes (to support re-purposing). KidneyGPS adopts a comprehensive approach summarizing evidence for all 5906 genes in the 424 GWAS loci for eGFR identified previously and the 35,885 variants in the 99% credible sets of 594 independent signals. KidneyGPS enables user-friendly access to the abundance of information by search functions for genes, variants, and regions. KidneyGPS also provides a function (“GPS tab”) to generate lists of genes with specific characteristics thus enabling customizable Gene Prioritisation (GPS). These specific characteristics can be as broad as any gene in the 424 loci with a known kidney phenotype in mice or human; or they can be highly focussed on genes mapping to genetic variants or signals with particularly with high statistical support. KidneyGPS is implemented with RShiny in a modularized fashion to facilitate update of input data (https://kidneygps.ur.de/gps/).

**Conclusion:**

With the focus on kidney function related evidence, KidneyGPS fills a gap between large general platforms for accessing GWAS and post-GWAS results and the specific needs of the kidney research community. This makes KidneyGPS an important platform for kidney researchers to help translate in silico research results into in vitro or in vivo research.

**Supplementary Information:**

The online version contains supplementary material available at 10.1186/s12859-023-05472-0.

## Background

Genome-wide association studies (GWAS) have successfully identified genetic loci associated with complex traits [1]. Human genetic evidence has been shown to improve the success rate in drug discovery, especially when the drug target is supported by a Mendelian trait or a GWAS association linked to a missense variant or an otherwise deleterious coding variant [2].

Chronic kidney disease is one of the top 15 causes of death in industrial countries and a poses a large individual and public health burden [3]. Impaired glomerular filtration rate (GFR) < 60 ml/1.73m^2^/min is a hallmark of CKD. GWAS have identified hundreds of genetic loci associated with GFR estimated from serum creatinine (eGFR) in the general population [4, 5]. However, linking the significant GWAS loci to causal variants and genes pinpointing molecular disease mechanisms relevant for kidney function is challenging.

Post-GWAS fine-mapping of identified loci aims to identify independent signals within loci and to narrow down each signal to the genetic variants that are likely the driving variant of an association signal (e.g., 99% credible sets of variants containing the causal variant with 99% probability, [6]). A 99% credible set of variants is generated for each signal. However, only the signals narrowed down to a limited number of variants in the credible set (e.g., small sets of five or less variants), or sets of any size that include a variant with a high posterior probability of association (e.g., PPA ≥ 50%), might be considered to have strong statistical support for pinpointing the rather likely causal variants. A signal with one variant in the credible set can be considered as narrowed down to single variant resolution: this variant has a 99% probability to be the causal variant for the association signal, given the causal variant is among the analysed variants. Such variant prioritizations have been adopted by Mahajan and colleagues (for Type 2 Diabetes) [7], Wuttke and colleagues (for kidney function) [5] and by Fritsche and colleagues (for Age-related macular degeneration) [8]. Another post-GWAS aim is then the mapping of the statistically supported genetic variants to genes by a variant’s predicted effect on the protein or regulatory function in relevant tissue. The relevant annotation data will typically depend on the disease or phenotype of interest.

The kidney researcher will typically want to focus on kidney tissue, if gene regulatory information is accessed, but also to have a look at other tissue to see whether gene regulation is kidney-specific or ubiquitous. A kidney researcher might also want to see whether a gene has a kidney phenotype in mice or human, or whether the gene is already a drug target in registered clinical trials for kidney disease. Yet, a gene targeted by clinical trials for other diseases might also be interesting for potential re-purposing. Thus, the relevant information should be kidney-centered, but not necessarily kidney-specific. A kidney researcher might also want to see genetic association evidence for kidney function biomarker other than creatinine-based eGFR, for eGFR in individuals with diabetes mellitus (DM), or for eGFR decline over time.

For a kidney researcher interested in selecting targets for functional follow-up or in judging pre-existing targets about GWAS support, the abundance of information from GWAS loci associated with kidney function and annotation data to map genes with kidney function relevance can be overwhelming. The data resulting from GWAS and post-GWAS is typically made publicly available either as online database or as supplementary tables of research articles. Supplementary tables are very specific, but cumbersome to search through. Online platforms like “GWAS catalog” [9], “Open Targets” [10] or “HugeAMP” [11] provide a broad view across numerous traits in parallel and integrate as many available GWAS results as possible. While this is comprehensive across traits, it can be challenging to extract the information relevant for a specific disease or trait. Bridging the gap between GWAS-based evidence and ready information for laboratory researchers is still an unmet need, particularly for kidney function genetics.

Our KidneyGPS, is a web-based application to query the evidence from GWAS results on eGFR joined with multiple annotation datasets and post-GWAS analyses that are relevant to the kidney. KidneyGPS also provides extended kidney-relevant genetic association results: (i) for alternative kidney function biomarkers, (ii) for eGFR in individuals with diabetes, and (iii) for eGFR decline. KidneyGPS can thus help the kidney-interested laboratory researcher to obtain an overview of existing genetic association support of specific genes and variants and to generate lists of genes with specific characteristics (customizable Gene PrioritSation, GPS).

## Construction and content

**Fig. 1:**
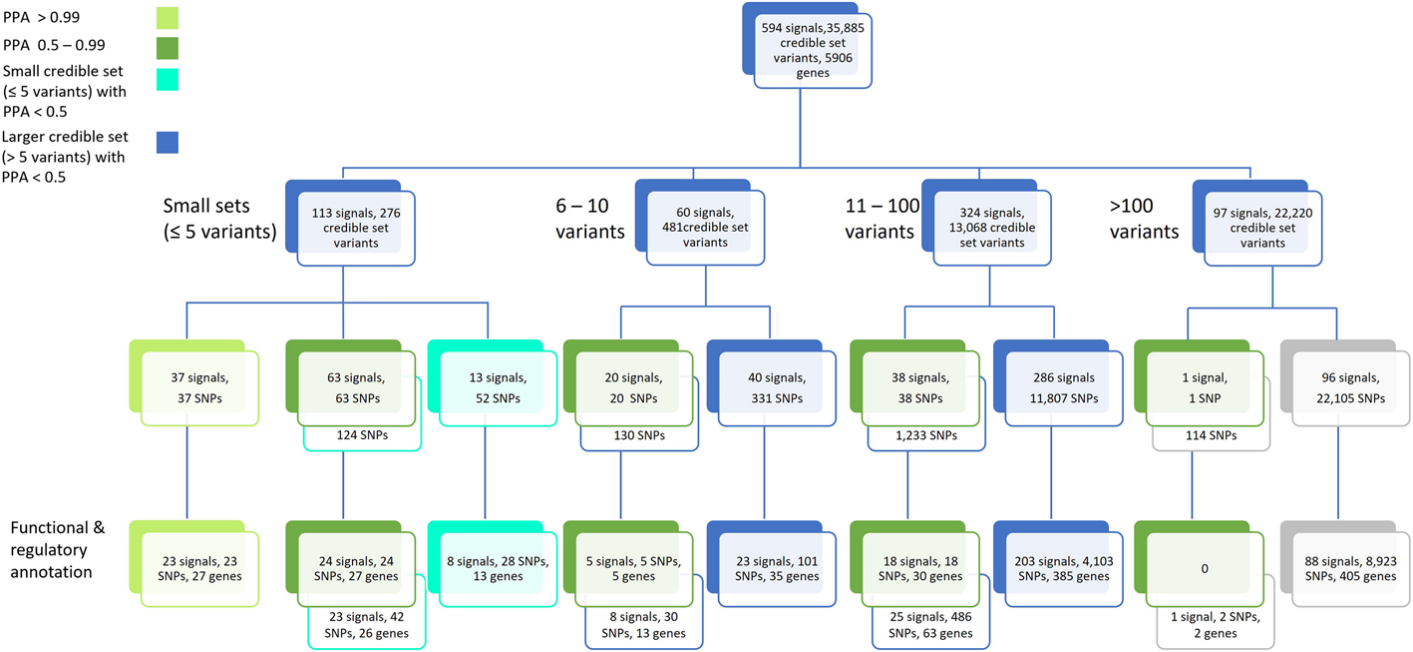
The 594 eGFR signals by strength of statistical support of pinpointing the likely causal variants. The 594 independent eGFR association signals were separated by size of their 99% credible set, stating the number of signals and number of variants in the corresponding 99% credible sets. In the 3rd row, we distinguished signals further by the highest PPA of a variant in a set: (i) one variant in the credible set (i.e. > 99% PPA, light green), (ii) at least one variant with PPA 50%-99% (dark green), or (iii) small credible set with all variants’ PPA < 50% (turquoise). Blue and turquoise boxes under dark green boxes contain the remaining variants in signals with one high PPA variant. The last row shows the number of respective variants that were mapped to a gene (protein-relevant or eQTL/sQTL in kidney tissue), also stating the number of mapped genes and number of signals with at least one credible set variant mapped to a gene

The primary backbone of KidneyGPS are the 424 loci identified from GWAS on eGFR from the Chronic Kidney Disease Genetics (CKDGen) consortium, the largest consortium dedicated to genetic research on kidney function, and its expansion by UK Biobank totalling 1,201,909 individuals of primarily European ancestry [4]. Each locus was fine-mapped to identify independent signals within the locus using approximate conditional analyses (GCTA [12]); for each signal, the 99% credible set of variants was generated using Bayesian fine-mapping [13] (i.e., smallest set of variants that contains the causal variant with 99% probability). Due to the lack of an appropriate trans-ethnic linkage disequilibrium (LD) reference panel (required for the GCTA analysis), the GCTA and Bayesian fine-mapping analyses were based on Europeans-only (i.e., on eGFR summary statistics from 1,004,040 Europeans and on a LD reference panel that consisted of 20,000 unrelated Europeans from the UK Biobank). Given the recently expressed concern regarding the limited robustness of fine-mapping results upon different rounding of GWAS association statistics [6, 14], we conducted sensitivity analyses. We found some of the signals and credible sets observed in the original analysis [4] as less robust when increasing the number of decimal digits in the per-variant association statistics from 4 to 6, but no relevant change when further increasing to 8 decimal digits (Additaional file 1: Note S1, Figs. S1, S2). We thus updated the fine-mapping results (independent signal identification, credible sets) using 6 decimal digits for the association statistics yielding 594 independent signals across the 424 loci with a total of 35,885 variants in the 99% credible sets. Among the 594 signals, 172 signals showed strong statistical support for pinpointing likely causal variants by a 99% credible set that was small (≤ 5 variants) or contained a high-PPA (≥ 50%) variant (Fig. 1). Comparison of variants’ PPA with the size of the credible set that contained the variant supported the idea that small credible sets were enriched for high PPA variants (Additaional file 1: Fig. S3).

For each of the 35,855 credible set variants, we queried annotation data to infer whether variants were protein-relevant or regulatory for any of the 5906 genes located the 424 eGFR loci (“variant-to-gene mapping”): (1) whether the variant resided within a gene and was protein-relevant by any of the three categories (using Ensembl Variant Effect Predictor, VEP [15], and Combined Annotation Dependent Depletion, CADD, [16] for the categories): (i) “stop-gained”, “stop-lost”, “non-synonymous (providing CADD-Phred score) (ii) “canonical-splice”, “noncoding-change”, “synonymous”, “splice-site” (providing CADD-Phred score) and (iii) other consequences restricted to CADD-Phred score ≥ 15 to ascertain protein-relevance; (2) whether the variant had an association with gene expression (eQTL, false discovery rate < 0.05) in kidney-tissue (tubulo-interstitium or glomerulus from NEPTUNE [17] or Susztaklab [18], kidney cortex from GTEx v7 [19]) or any tissue (GTEx v7); (3) whether the variant was associated with expression levels of exon junctions or variation in the relative abundances of gene transcript isoforms (splice quantitative trait locus, sQTL, FDR < 0.05) in any tissue (GTEx V7).

We also queried annotation data for the 5,906 genes (“gene-to-phenotype mapping”): (4) whether the gene had kidney-relevant phenotypes in mice (Mouse Genome Informatics, MGI [20]); (5) whether the gene was known for human genetic disorders with kidney phenotype (Online Mendelian Inheritance in Man, OMIM, [21]) or with evidence from sequencing patients (CKD patients [22] or autosomal dominant tubulointerstitial kidney disease patients [23]); (6) whether the gene was known for drugability or drug-interaction from registered clinical trials (Therapeutic Target Database [24]) for kidney-related indications (ICD-11 codes GB4“X” to GB9“X”, Additional file 1: Table S1), also providing the information on other indications (relevant for re-purposing).

Additionally, for each of the 594 eGFR signals, we queried further genetic association data relevant to the kidney researcher: (7) To highlight the relevance of a genetic association with creatinine-based eGFR for kidney function rather than creatinine metabolism, we included information on whether the locus association was directionally consistent and nominally significant for blood urea nitrogen (BUN) or cystatin-based eGFR (eGFRcys; i.e. locus lead variant *P* < 0.05; opposite or same direction of effect for BUN or eGFRcys, respectively; n = 852,678 or 460,826, respectively; yielding 491 of 594 signals validated); (8) Since genetic effects with steeper decline versus more stable eGFR over time might point to particularly deleterious mechanisms for the kidney, we included information on whether the signal showed significant association on eGFR decline (N = 343,339 [25], yielding 8 decline signals). (9) Since eGFR etiology in DM might differ from eGFR generally, we included information on the eGFR association for DM and noDM individuals separately and whether the eGFR signals were more or less pronounced in DM versus noDM (N_DM_ = 178,691, N_noDM_ = 1,296,113 [26], yielding 6 signals associated with eGFR only or more strongly in DM and 1 signal only associated in noDM).

 The specific aspects and annotation data integrated in KidneyGPS as of now (version 2.3.0) and how this compares to the previous publication [4] are shown in Additaional file 1: Table S2 and the KidneyGPS online documentation. By these annotations (of the 35,855 variants, 5906 genes), KidneyGPS contains (a) 13,716 credible set variants that mapped to any of 940 genes as being protein-relevant or eQTL/sQTL in kidney tissue (Table 1A) and (b) 381 genes with known kidney phenotype in mouse or human (including drug targets for kidney diseases; Table 1B).

**Table 1:** Annotation of credible set variants and genes in the 594 signals associated with eGFR

A				
Annotation feature	PPA > 99% (# sig | #var | #genes)	PPA 50–99% (# sig | #var | #genes)	other variants in small sets (# sig | #var | #genes)	any other variants (# sig | #var | #genes)
Protein-relevant (all, VEP)	14 | 14 | 14	22 | 22 | 22	15 | 18 | 16	279 | 950 | 520
Stop-gained/-lost; non-synonymous	10 | 10 | 10	16 | 16 | 16	6 | 6 | 6	127 | 271 | 197
Canonical splice, noncoding change, synonymous, splice site	1 | 1 | 1	2 | 2 | 2	2 | 2 | 2	147 | 342 | 247
Other (CADD-Phred ≥ 15)	3 | 3 | 3	4 | 4 | 4	9 | 10 | 9	169 | 344 | 217
eQTL kidney tissue (all)	11 | 11 | 14	28 | 28 | 41	24 | 61 | 28	239 | 13,025 | 520
Tubulo-interstitium	10 | 10 | 11	22 | 22 | 30	21 | 54 | 23	205 | 10,472 | 400
Glomerulus	7 | 7 | 10	18 | 18 | 26	15 | 38 | 16	195 | 10,562 | 362
Kidney cortex	0 | 0 | 0	2 | 2 | 2	1 | 1 | 1	30 | 1,267 | 36
sQTL kidney cortex	0 | 0 | 0	0 | 0 | 0	1 | 1 | 1	13 | 522 | 23
Any of the above	23 | 23 | 27	47 | 47 | 61	31 | 70 | 39	348 | 13,576 | 865

B	
Feature (source)	# genes
Genes with kidney phenotype in human (all sources)	235
Online Mendelian Inheritance in Man (OMIM) genes	163
Genes from sequencing CKD Patients (Groopman et al*.*, 2019)	178
Genes from sequencing ADTKD patients (Wopperer et al., 2022)	12
Genes with kidney phenotype in mouse models (MGI)	342
Gene is drug target in registered clinical trial (TTD)	499
By trial with kidney disease indication	7
By trial with any other indication	492
Any of the above	866

The 35,885 variants in the 99% credible sets were queried for being protein-relevant or eQTL/sQTL in kidney tissue to any of the 5,906 genes; the 5,906 genes were queried for kidney phenotypes in human or mouse, and for drugability. A: Separating the variants by strength of statistical support, we show the number of variants that are protein-relevant [11, 16] or an eQTL/sQTL [17–19] in kidney tissue, the number of signals, and the number of mapped genes. B: Shown are the number of genes known for (i) causing a genetic disorder in human with kidney phenotype [21–23], (ii) a kidney phenotype in mouse [20] (iii) being drug target in registered clinical trials for kidney disease or any other diseases [24]

Columns: “PPA > 99%”: exactly one variant in credible set; “PPA 50–99%”: variant has high PPA; “other variants in small set”: variant in small set that has PPA >  < 50%; “any other variants”: variants with PPA >  < 50% and set size > 5); Abbreviations: VEP: Variant effect predictor, CADD-Phred: Score for deleteriousness of a variant, eQTL: expression quantitative trait locus, sQTL: splice quantitative trait locus, #sig: number of signals, #var: number of variants in 99% credible sets

*CKD* chronic kidney disease, *ADTKD* autosomal dominant tubulointerstitial kidney disease, *MGI* mouse genome informatics, *TTD* therapeutic target database

**Fig. 2:**
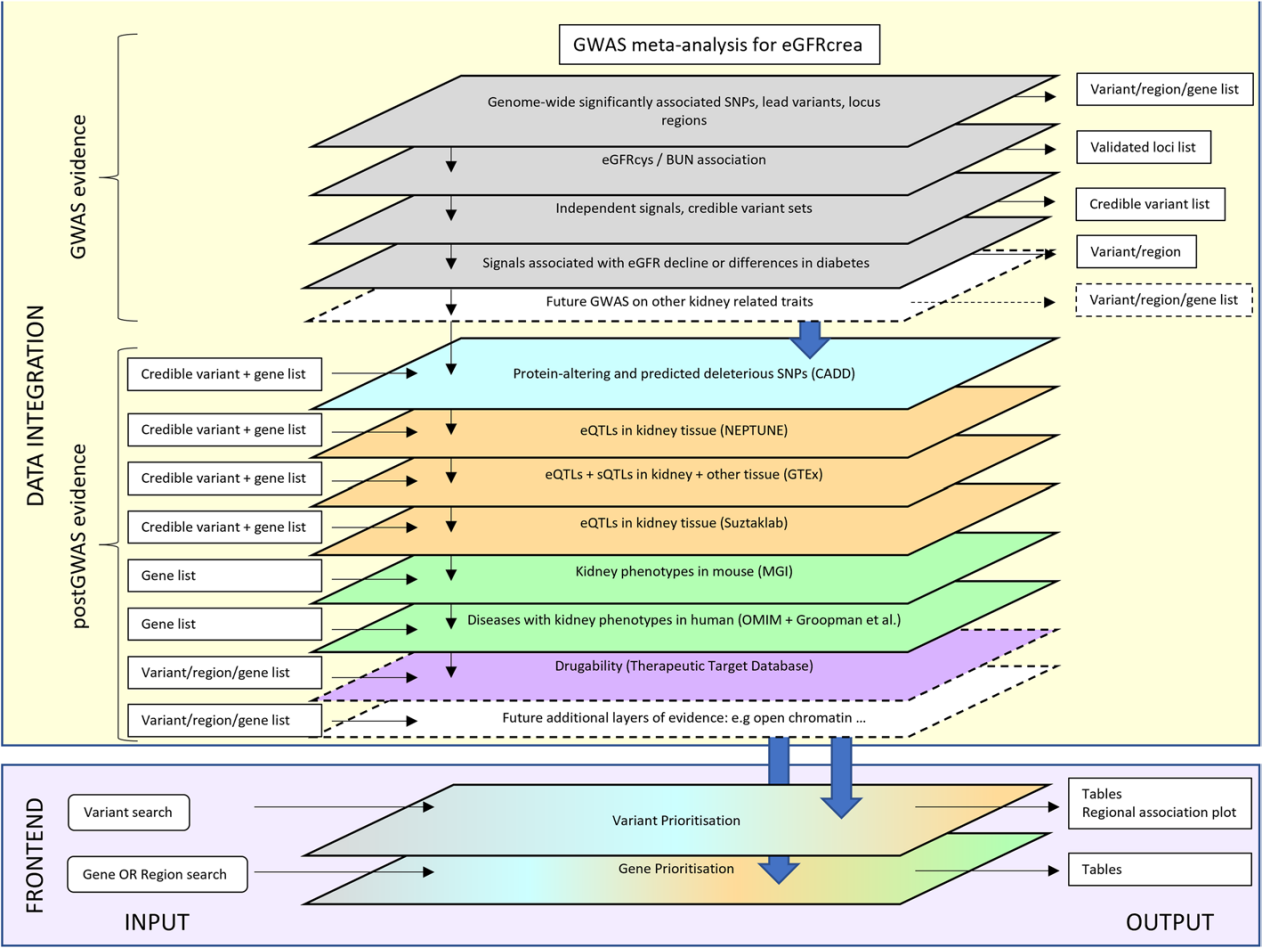
Overview of the architecture of KidneyGPS. We integrated data from multiple GWAS and post-GWAS approaches and derived evidence for variant and gene prioritization regarding their impact on kidney function. Shown are the integrated datasets by the various levels (with the respective input and output of the query within the software) and the front end (input and output of the query of the user); implemented as web application using RShiny

The architecture of the KidneyGPS database is structured hierarchically based on the 424 eGFR loci and annotation data, where each of these above outlined steps are considered additional layers of evidence that are partly locus-specific, signal-specific, variant-specific, or gene-specific (Fig. 2). KidneyGPS is implemented as RShiny web application (https://kidneygps.ur.de/gps/).

## Utility and discussion

### User interface

The user interface of KidneyGPS is organized into five tabs: Three tabs enable the specific search for genes, variants and regions (underlying data structure shown in Additional file 1: Fig. S4): (1) “gene search” tab: search for genes using their gene names (synonyms automatically mapped to their official HGNC gene name [18]), (2) “variant search” tab: search variants using their rs-identifiers or genetic positions (chr:pos from GRCh37), and (3) “region search” tab: search for start and end of a genomic regions using chromosome and base-positions. (4) The GPS tab enables to generate lists of genes with specific characteristics and thus enables customized gene prioritization. (5) The last tab, labelled “Documentation & Help”, includes step-by-step-guides for search and GPS features, data source descriptions, and contact options, and data privacy statement.

The GPS tab summarizes the integrated data on all 5906 genes and 594 signals in a comprehensive “GPS Table”. It enables gene prioritization by generating lists of genes with specific characteristics, using various filter options: for example, all genes in the 594 eGFR signals that have a known kidney phenotype in mice or human, or all genes mapping to signals with specific properties regarding the strength of statistical support for pinpointing the likely causal variant. There are three types of filter options: (1) “*Signal filtering”* allows to restrict the view of the GPS-table to signals with small credible sets (≤ 5 variants) or to signals with a high PPA variant in the credible set (10%, 50% or 99%) or any combination of the two filters. Additionally, one can restrict the GPS-view to signals where the genetic association with creatinine-based eGFR is eGFRcys/BUN-validated, modulated in individuals with DM, or with a genetic association also for eGFR decline over time. (2) “*Variant-to-gene mapping”* allows to further restrict the GPS-view to genes that are mapped by credible set variants with specific characteristics, e.g., protein-altering variants, or regulatory variants in kidney tissue. (3) “*Gene-to-phenotype mapping*” allows to restrict the GPS-view to genes of specific characteristics, e.g., known for human or mouse kidney phenotypes, or for genes that are drug targets in registered trials for kidney disease. Case examples of how to use the GPS tab are provided in Additional file 1: Note S2.

The “gene search” tab enables the user to search for up to 2,000 genes simultaneously, either as a list or a text-file input. The generated output provides an extract of the GPS-Table for the respective gene(s) as well as all underlying gene- and variant-based information. The variant-based information can be restricted to variants with PPA above a user-defined threshold. The “variant search” is similarly structured allowing for simultaneous querying of up to 2000 variants. The output yields the eGFR association statistics (all ancestries) for any variant that was genome-wide significantly associated with eGFR (*P* < 5 × 10^–8^). For credible set variants (regardless of their association *p* value), it yields eGFR association statistics in European-ancestry (with and without fully conditioning on independent signal index variants of the locus) and functional or regulatory annotation, if applicable. The “region search” enables the user to identify eGFR associated loci that overlap their genomic region of interest and provides the GPS Table for all genes within these loci. For the gene and variant search, we also provide regional association plots of the respective eGFR locus (LocusZoom [27]). All search results are available for download as Excel tables or as plain csv files.

### Limitations and potential misinterpretations

Understanding the limitations of the methods and data employed in KidneyGPS is crucial to avoid misinterpretations.

There is some uncertainty in the robustness of independent signal identification and generated credible sets of variants [6]. The signal identification uses the variants’ correlations based on variant reference panels. Due to the lack of an appropriate trans-ethnic reference panel, here, a reference panel of 20,000 unrelated Europeans from UK Biobank was used, which fits very well to the integrated UK Biobank GWAS, but with some uncertainties regarding its fit to other European or non-European GWAS studies included in CKDGen. The generation of credible sets of variants for each signal was performed based on European-only eGFR summary statistics using the method by Wakefield [13], which assumes one causal variant per signal and thus depends on successfully distinguishing the independent signals up front. Alternative approaches allowing for multiple causal variants per signal like SuSiE [28, 29] or FINEMAP [30] are currently investigated, but have not been widely applied to large consortia data so far and might even more strongly depend on the variant reference panel and power. Any approach for generating credible sets of variants will successfully narrow down only a part of all GWAS signals due to power and regions with large stretches of jointly inherited variants (i.e., high LD). Credible sets as wide as > 100 or even > 1000 genetic variants or credible set variants with low PPA provide limited credibility in the statistical support of any of these variants and users might want to use the “signal filter” of KidneyGPS to focus on signals and/or variants with high statistical support. Currently emerging methods to investigate fine-mapping robustness, e.g., SLALOM [14], might help quantify the uncertainties in fine-mapping results. Integrating more individuals of African ancestry can help dissect large stretches of high LD in the genome and sharpen GWAS signals in the future.

It is a limitation that the GWAS association statistics for eGFR are based mostly and the fine-mapping solely on European populations. Therefore, KidneyGPS results cannot be generalized to non-European populations when genetic variant frequencies differ or when the causal variants even vary between ancestries. For example, multiple variants in *APOL1* have high impact on kidney function in African Americans [31], but are not associated in European ancestry due to low allele frequencies. Currently emerging larger GWAS on kidney function with improved coverage of non-European populations and emerging multi-population fine-mapping methods will provide the opportunity to expand KidneyGPS to represent individuals more globally.

With regards to the phenotype used for the GWAS, some genetic effects identified for creatinine-based eGFR may not necessarily impact kidney function but creatinine metabolisms. Our filter option for eGFRcys or BUN validated signals can be used to provide more certainty on kidney function relevance.

Finally, genetic effects on eGFR might differ by sex, in the presence of hypertension, or have other interactions with lifestyle factors, but GWAS within these subgroups or GWAS incorporating gene-lifestyle interactions are currently lacking. We do provide genetic association of eGFR in DM [26] and highlight the—surprisingly few—differences of these with general eGFR.

### Comparison with existing tools

While other post-GWAS tools like GWAScatalog [9], HugeAMP [11] and Open Target Genetics [10] are more comprehensive across diseases and traits, KidneyGPS stands out due to its focus on kidney function, its direct link to other kidney-related GWAS, and due to its customizable approach to generate lists of genes of specific characteristics (Table 2). Although all platforms provide variant- and gene-search functionalities, KidneyGPS outperforms the other platforms in terms of the depth of kidney-related results and the options available to extract kidney function specific information (real showcase example in Additional file 1: Note S3). KidneyGPS is unique in its customizable approach to gene prioritization in the sense that the user can generate lists of genes of specific characteristics depending on personal preference or research interest, rather than prioritizing genes by a specified algorithm that typically implies assumptions. An advantage of GWAS Catalog [9], Open Targets Genetics [10] or HugeAMP [11] is that users can extract information across many different phenotypes. However, due to the focus on data related to kidney function, KidneyGPS is a practical tool for the kidney researcher enabling easy access and easy information extraction.

**Table 2:** Feature comparison across GWAS online platforms

Feature	KidneyGPS	GWAS Catalog [9]	Open Targets Genetics [10]	HugeAMP [11]
*Focus on kidney function*				
eGFR associations [4]	y (until version 1.3.1)	y	n	y
eGFR associations (Stanzick et al. updated)	y	n	n	n
eGFR associations [32]	n	y	n	y
eGFR associations [5]	n	y	y (lead variants)	y
eQTL/sQTL data (kidney tissue)	y	n	Partly (in coloc)	n
BUN association [4]	For lead variants	y	n	y
eGFRcys association [4]	For lead variants	y	n	y
eGFR in DM versus noDM [26]	y	n	n	n
eGFR over time [25]	For lead variants	n	n	n
Genes with kidney phenotypes in mouse or human	y	Link to OMIM^c^	Link to open targets^c^	Link to MGI^c^
Genes targeted by kidney disease drugs	y	n	n	n
*Search functionality*				
Gene search	y	y	y	y
Variant search	y	y	y	y
Region search	y	y	n	y
*Gene prioritization*				
Machine learning derived gene scores	n	n	y	y
Filter options for gene prioritization	y	n	n	n
Prioritization per locus	y	n	y	n
Prioritization across loci	y	n	n	n
*Features*				
Protein-relevant variants	y	Partly^b^	Integrated but not extractable	y
eQTL data (any tissue)	y	n	Integrated but not extractable	Integrated but not extractable
sQTL data (any tissue)	y	n	n	n
Genes with kidney phenotypes in mouse or human	y for kidney	Link to OMIM	Link to open targets^c^	Link to MGI
Drug target information	y	n	Link to open targets^c^	n
Genomic and epigenetic features ^a^	Planned	n	y	y
Phewas	Planned	n	y	y
pathway analyses	Planned	n	Link to open targets	y

We compared KidneyGPS with three other post-GWAS platforms. We contrasted search functionalities, approaches to gene prioritization (machine learning derived gene scores or customizable prioritization via filter options to generate lists of genes with specific characteristics) and integrated features

^a^Binding sites, open-chromatin, enhancer, promotor

^b^Information only available when searched for 1 variant (not downloadable, not for lists of variants in a searched region)

^c^No focus on kidney phenotype, DM: Diabetes mellitus

### Future developments

We have developed KidneyGPS within a cooperation with the CKDGen consortium and we will continue to cooperate there. This will enable timely updates with novel loci and signals for eGFR, more diverse populations included in the GWAS, improved resolution at already existing signals, and further expansion to other kidney function traits or subgroup-specific GWAS. Other updates will include information on open chromatin in kidney tissue, kidney cell-type specific gene expression and enriched pathway.

## Conclusion

KidneyGPS summarizes the statistical and biological evidence identified by GWAS for eGFR and post-GWAS approaches with a special focus on kidney-relevant annotation data as well as complementing genetic associations for other kidney-related traits. KidneyGPS displays this information in a clear and comprehensive way to make it easily accessible to a broader interdisciplinary scientific community. KidneyGPS is designed to support kidney researchers to help translate in silico research results into in vitro or in vivo research.

### Supplementary Information

**Additional file1**: **Note S1**. Sensitivity Analyses regarding the impact of decimal digits on secondary signal identification and fine-mapping results. **Note S2**. Case example on how to use the “GPS tab”. **Note S3**. Detailed comparison of gene-search and results from KidneyGPS to GWAS Catalog, Open Targets Genetics and HugeAMP. **Table S1**. ICD-11 codes. **Table S2**. Comparison of data. **Fig. S1**. Comparison of the identifying p-values of the 634 signal index variants in analyses with different numbers of digits analysis. **Fig. S2**. Comparison of credible set sizes for all 634 signals. **Fig. S3**. Comparison of variant’s PPA and 99% credible set size. **Fig. S4**. Architecture of KidneyGPS search functions.

## Data Availability

The KidneyGPS can be accessed at https://kidneygps.ur.de/gps/ and the source code is available at https://github.com/kjstanzick/KidneyGPS. KidneyGPS integrates data from various sources of information including kidney function loci [4] gene expression data [17–19], functional annotation and CADD [15, 16], kidney phenotypes [20–23], diabetes-specific kidney function [26], kidney function decline [24, 25].
