# Supplementary material for: KidneyGPS: a user-friendly web application to help prioritize kidney function genes and variants based on evidence from genome-wide association studies
Source: BMC Bioinformatics. 2023 Sep 21;24:355. doi: 10.1186/s12859-023-05472-0 (PMC10512588; doi:10.1186/s12859-023-05472-0)
Supplement: Supplementary file 1 — Additional file1: Note S1. Sensitivity Analyses regarding the impact of decimal digits on secondary signal identification and fine-mapping results. Note S2. Case example on how to use the “GPS tab”. Note S3. Detailed comparison of gene-search and results from KidneyGPS to GWAS Catalog, Open Targets Genetics and HugeAMP. Table S1. ICD-11 codes. Table S2. Comparison of data. Fig. S1. Comparison of the identifying p-values of the 634 signal index variants in analyses with different numbers of digits analysis. Fig. S2. Comparison of credible set sizes for all 634 signals. Fig. S3. Comparison of variant’s PPA and 99% credible set size. Fig. S4. Architecture of KidneyGPS search functions. [file 12859_2023_5472_MOESM1_ESM.docx]

**Supplementary Material**

**Contents**

[SUPPLEMENTARY NOTE 2](#_Toc142073636)

[Supplementary Note 1: Sensitivity Analyses regarding the impact of decimal digits on independent signal identification and fine-mapping results. 2](#_Toc142073637)

[Supplementary Note 2: Case example on how to use the “GPS tab” 3](#_Toc142073638)

[Supplementary Note 3: Detailed comparison of gene-search and results from KidneyGPS to GWAS Catalog, Open Targets Genetics and HugeAMP 4](#_Toc142073639)

[SUPPLEMENTARY TABLES 8](#_Toc142073640)

[Supplementary Table 1: ICD-11 codes for kidney disorders in registered clinical trials 8](#_Toc142073641)

[Supplementary Table 2: Comparison with previous work 9](#_Toc142073642)

[SUPPLEMENTARY FIGURES 10](#_Toc142073643)

[Supplementary Figure 1: Comparison of the identifying p-values of the 634 signal index variants in analyses with different numbers of digits analysis 10](#_Toc142073644)

[Supplementary Figure 2: Comparison of credible set sizes for all 634 signals. 11](#_Toc142073645)

[Supplementary Figure 3. Comparison of variant’s PPA and 99% credible set size. 12](#_Toc142073646)

[Supplementary Figure 4: Architecture of KidneyGPS search functions. 13](#_Toc142073647)

[References 15](#_Toc142073648)

# **SUPPLEMENTARY NOTE**

## **Supplementary Note 1: Sensitivity Analyses regarding the impact of decimal digits on independent signal identification and fine-mapping results.**

Previously, Stanzick and colleagues conducted a genome-wide association study (GWAS) meta-analysis on glomerular filtration rate estimated from serum creatinine (eGFRcrea) using data from the Chronic Kidney Disease Genetics (CKDGen) consortium and UK Biobank (UKB) with default settings for METAL, resulting in effect estimates and standard errors with 4 decimal digits. Approximate conditional analysis with GCTA identified 210 secondary signals yielding a total of 634 independent eGFR signals within the 424 eGFR GWAS loci. To assess the robustness of these signals, we conducted re-analyses with increased decimal digits (6/7 and 8/8 for effect estimates and standard error) and compared the results to the original analysis. We adjusted the METAL settings for the meta-analysis of CKDGen and UKB to obtain association statistics with 6/7 or 8/8 decimal digits. We then performed approximate conditional analyses for the 6/7 and the 8/8 meta-analyses with GCTA, using the same reference panel of 20K unrelated Europeans from UKB, which had been used in the work by Stanzick and colleagues. The original 634 signal index variants (identified by the step-wise conditional approach in Stanzick et al.) were used as fixed list of variants to stepwise condition on (further referred to as identifying analysis) and to condition one signal on all other signals in the same locus (further referred to as fully conditioning analysis). Based on the fully conditioned results we calculated approximate Bayes factors and posterior probabilities of association (PPA) for each variant in each signal with the Wakefield method as done previously. 99% credible variant sets for each of the 634 signals were obtained by adding the variants with the highest PPA to the set until a cumulative PPA >99% was reached.

By comparing the identifying p-values of the 210 secondary signal index variants between analyses with different decimal digits, we found that changing from 4/4 to 6/7 decimal digits altered the p-values of 67 out of the 210 secondary signals in a way that these variants were no longer genome-wide significant (GCTA conditional P> 5*10^-8^, **Supplementary Figure 1 A+B**). However, comparing the identifying p-values of the 6/7 decimal digits analysis with the 8/8 decimal digits analysis showed nearly identical results (**Supplementary Figure 1C**). This indicated that the increase from 6/7 to 8/8 decimal digits did not affect signal stability. A comparison of fine-mapping results between analyses of different decimal digits yielded similar results. The 99% credible set size differed substantially between the 4/4 and the 6/7 decimal digits analysis but remained comparable between the 6/7 decimal digits and 8/8 decimal digits analyses (**Supplementary Figure 2**).

Based on these results, we repeated the independent signal identification approach (stepwise conditioning with GCTA without pre-defined index variants) using the 6/7 decimal digits data set, resulting in 170 secondary signals instead of the initial 210. Among the 210 and 170 index variants, 61 were identical, 35 were highly correlated (r^2^ >0.9; based on UKB), 22 were moderately correlated (r^2^ between 0.5 and 0.9) and 92 or 52 index variants of the 4/4 decimal digits and 6/7 decimal digits analyses respectively, were not comparable.

## **Supplementary Note 2: Case example on how to use the “GPS tab”**

The GPS tab of KidneyGPS can provide customized lists of ‘prioritized’ genes. It comprises three filter sections that are applied to the full and comprehensive GPS table (5,906 genes for 594 signals), which is shown by default: the first section, called “Signal-Filtering”, enables to restrict the full GPS-table to signals with specific properties, such as statistical fine-mapping support or Cys/BUN-validation of the signal; the second section, called “Variant-to-Gene mapping”, enables to further restrict to genes that are mapped by variants with specific properties, such as protein-relevance or regulatory variants; and the third section, called “Gene-to-phenotype mapping”, enables to further restrict to genes with known kidney phenotype in mice, human or to druggable genes. To demonstrate how to use these filters, we will exemplary show how to

(1) generate a list of genes that are mapped to a signal with high statistical fine-mapping support for pinpointing the causal variant (credible set contains ≤5 variants or contains one variant with PPA≥50%), where the variant is protein-altering (using variant-effect-predictor; VEP) or an eQTL/sQTL in kidney tissue,

(2) further restrict this list to genes with evidence of a kidney phenotype in human (e.g. OMIM) or with evidence of being druggable for any disease, and

(3) further restrict to genes where the genetic effect is stronger in individuals with diabetes compared to individuals without diabetes.

To obtain the first gene-list (No. 1):

- In the first “Signal-filtering” section, we set “The signal's credible set contains a variant with posterior probability of association (PPA) of: 50%” and “signal has a small credible set (1-5 variants)”. This will restrict the full GPS-table to genes located in signals satisfying at least one of those criteria.
- In the second section “Variant-to-gene mapping”, we must then select all blue options and the orange options “eQTL in glomerular tissue”, “eQTL in tubulo-interstitial tissue”,” eQTL in kidney cortex tissue”, and “sQTL in kidney cortex tissue”. This will further restrict our list of genes to genes mapped by a credible set variant that is protein-altering or by a credible set variant that is an eQTL or sQTL in kidney tissue. If any blue or orange feature is chosen, you can further restrict the mapping based on the strength of statistical support of the mapped variant itself. The default is that the mapped variant must have a PPA > 50% or must be included in a small credible set, which fits with our request. Thus, we can click on “Go prioritize!” to generate our first gene-list. The displayed GPS-Table is now reduced to genes which meet our applied criteria. To save this list, we can use the “Download Results“ button, which will store the list as an Excel file. Below the download button, we can see that our list included 110 different genes from 86 independent eGFR signals.

To further restrict the list of genes to genes with evidence of a kidney phenotype in human (e.g., OMIM) and/or with evidence of being druggable for any disease (No. 2):

- In the “Gene-to-phenotype mapping” section, the second option of “Kidney phenotypes” is required to restrict to genes with evidence of a kidney phenotype in human.
- Additionally, both options of “Drug Information” must be selected to show genes that are drug targets of any disease.
- After applying these three selections and again clicking “Go prioritize!”, the GPS-Table view is further restricted to 21 genes from 20 signals.

To generate the 3rd gene list (No. 3), we provide two options:

- The first one is to use the table header filter possibility for the “Signal association depends on DM” column and select the “DM>NoDM” option. This will show us that only the *UMOD* gene meets all our pre-defined criteria.
- Alternatively, in the “Signal filtering” section, one can use the “Restrict further to: Signals with differential association in individuals with diabetes and those without diabetes” option in the “Signal-Filtering” section. After clicking “Go prioritize!”, the GPS-Table view is restricted to *UMOD*. The latter approach is recommended for download because the filtering options in the header line of the GPS-table are not preserved in the downloaded data set (one would have to open the file in Excel and re-apply the filtering in Excel).

## **Supplementary Note 3: Detailed comparison of gene-search and results from KidneyGPS to GWAS Catalog, Open Targets Genetics and HugeAMP**

To highlight the distinctive features and advantages of our KidneyGPS, we conducted a comparative analysis of its gene-search feature with three other platforms, namely GWAS Catalog [1], Open Targets Genetics [2] and HugeAMP [3] . Each platform offers a gene-search functionality on its main page, but our platform stands out as the sole one that allows users to restrict search results based on specific features. To demonstrate the utility of our platform, we used *SLC47A1*, a gene recently linked to kidney disease [4]), as an exemplary gene.

The results of GWAS Catalog, Open Targets Genetics and HugeAMP commence with a general gene information. GWAS Catalog and Open Targets provide details on the corresponding protein name ("solute carrier family 47 member 1"), genomic location (GRCh38: 17:19,495,385-19,579,034), biotype (protein coding), and links to other platforms describing the gene. GWAS Catalog links to NCBI (http://www.ncbi.nlm.nih.gov/), Ensembl (https://www.ensembl.org/Homo_sapiens/), Open Targets [5], OMIM (online mendelian inheritance in men) [6] and IMPC (International mouse phenotyping consortium) [7], while Open Targets Genetics refers to Ensemble (https://www.ensembl.org/Homo_sapiens/), gnomAD [8], GTEx and GeneCards [9] . In contrast, the gene summary of HugeAMP includes alternative gene names, genomic position and a functional summary extracted from Uniprot (https://www.uniprot.org/). Moreover, Gene Ontology and pathway annotations are available in HugeAMP.

GWAS Catalog provides, beside the gene information, 53 reported associations (variant level) mapped to *SLC47A1*, along with information on the 44 studies that identified these associations and a list of 16 traits displaying such associations. To extract the kidney function related information, users must identify the corresponding traits (e.g., glomerular filtration rate and chronic kidney disease) and use them as filters for the associations. Besides associations from other GWAS studies this also leads to the signal index variants of the GWAS data integrated in KidneyGPS. However, no further functional information at the variant-level is provided.

Similarly, Open Targets Genetics presents a table of studies that found associations of variants located within or near *SLC47A1*, encompassing 159 entries reflecting various traits, along with association statistics of the respective lead variant. One of the first entries belongs to glomerular filtration rate in a study from 2016 [10]. Notably, Open Targets Genetics lacks the study on which KidneyGPS is based on [11]. Yet, Open Targets Genetics incorporates a gene’s L2G pipeline score, a machine learning-derived gene prioritization score, which offers a link to the gene prioritization results of the locus where the searched gene is located in. For *SLC47A1* and the glomerular filtration rate study from 2016, this score is 0.8, which is the highest compared to the other genes in this locus. While the partial L2G scores of the different features are listed (Variant Pathogenicity, Distance, QTL Coloc and Chromatin Interaction), the specific variants integrated into the score are not apparent. Furthermore, Open Targets Genetics provides information on molecular traits and other GWAS studies colocalizing with the respective locus but lacks QTL data from kidney tissue.

HugeAMP presents a more graphical search results page. Following the general gene information, it displays Gene-level associations for *SLC47A1* based on internal HuGE Scores that quantifies the genetic support for involvement of a gene in diseases or traits. A graphical representation is used to highlight traits with high HuGE Scores, which combine common and rare variant associations. For *SLC47A1*, the HuGE Scores of serum creatinine and eGFRcrea rank highest, with the "evidence range" being labeled as compelling. Subsequent sections provide information on tissue-specific gene expression for *SLC47A1*, including kidney-specific mRNA level measurements in different cell types. However, the information is sorted in alphabetic order, making it difficult to compare and interpret the reported values. Another section lists five studies reporting *SLC47A1* as predicted effector gene, including the study on which KidneyGPS is built on [11]. Lastly, UniProt cross-references are listed. While this main results page lacks variant-based association statistics or functional information, users can access the gene region page via the "Explore Region" button. There, the most significant associations in the region, including association statistics of lead variants (and LD proxies) for different phenotypes, are presented. Additionally, the Genome Region Miner allows users to select phenotypes of interest, providing an interactive locus zoom plot and all association statistics for all variants for the selected phenotype in the region, along with predicted functional consequences.

In contrast, KidneyGPS’s search results start with the Gene-Prioritisation (GPS) Table excerpt for *SLC47A1*, encompassing all selected features: GFR estimated from serum cystatin C (eGFRcys)/ blood urea nitrogen (BUN) validation of the association locus, signal interaction with diabetes mellitus (DM), signal association with eGFRcrea decline, the number of credible set variants mapped to the respective eGFRcrea signal, the maximal PPA of a respective credible set variant, information on any credible set variant being protein-alterering for *SLC47A1*, and whether any credible set variant is an eQTL or sQTL in kidney (or other) tissue, along with colocalization results of association with expression signals in kidney tissue, information on kidney phenotypes in mice or humans, whether the gene is a known drug target, and the genomic position (GRCh37). For *SLC47A1,* two rows are displayed in this GPS Table, describing two independent signals associated with eGFRcrea in the locus where *SLC47A1* is located. None of the two signals is validated with eGFRcys or BUN there is no interaction with DM status and no association with longitudinal eGFRcrea decline. The first signal contains one credible set variant with a PPA of 100% which is a stop-gained, stop-lost, or non-synonymous variant. The second signal contains 11 variants with a maximal variant-PPA of 32%. One of these 11 variants is an eQTL in glomerular tissue und 4 variants are eQTLs in tubulo-interstitial tissue. All 11 variants are eQTLs in other tissues from GTEx. Furthermore, the association signal of the second signal co-localizes with the expression signal in tubulo-interstitial tissue. A kidney phenotype is described in mice, but not in humans. Moreover, *SLC47A1* is a known drug target. More information on the missense variant, the eQTLs, the mouse phenotype, and the drugs interacting with *SLC47A1* can be found below the general GPS-Table in data excerpts showing detailed variant and gene-level results.

In summary, the four platforms offer distinct information when searching for *SLC47A1*. GWAS Catalog solely provides information on association statistics, with additional gene information accessible through links to other websites. Open Targets Genetics and HugeAMP offer comprehensive details on the gene, including machine-learning based individual gene scores and various functional information. While the other three platforms integrate information on different phenotypes, making the relevance for kidney function less evident, KidneyGPS stands out due to its focus on kidney function-related information, while being more comprehensive for this specific trait.

# **SUPPLEMENTARY TABLES**

**Supplementary Table 1:** **ICD-11 codes for kidney disorders in registered clinical trials**. We referred to the ICD-11 Browser of the WHO (https://icd.who.int/browse11/l-m/en) to identify the relevant ICD-11 codes for kidney diseases. This table shows the 31 codes used to separate kidney disease related drug indication from non-kidney disease related indications. Listed are the ICD-11 codes, the referring condition, and its parental term. We linked the kidney-disease ICD-11 codes to the ICD-11 codes available from the Therapeutic Target Database (TTD) to identify TTD drugs that are linked to kidney disease.

| ICD-11 code | Condition | Parental term |
| --- | --- | --- |
| GB40 | Nephritic syndrome | Glomerular diseases |
| GB41 | Nephrotic syndrome |  |
| GB42 | Persistent proteinuria or albuminuria |  |
| GB4Y | Other specified glomerular diseases |  |
| GB4Z | Glomerular diseases, unspecified |  |
| GB50 | Acute tubulo-interstitial nephritis | Renal tubulo-interstitial diseases |
| GB51 | Acute pyelonephritis |  |
| GB52 | Acute tubular necrosis |  |
| GB53 | Acute renal papillary necrosis |  |
| GB54 | Tubulo-interstitial nephritis, not specified as acute or chronic |  |
| GB55 | Chronic tubulo-interstitial nephritis |  |
| GB56 | Obstructive or reflux nephropathy |  |
| GB57 | Nephrocalcinosis |  |
| GB58 | Pyonephrosis |  |
| GB59 | Renal or perinephric abscess |  |
| GB5Y | Other specified renal tubulo-interstitial diseases |  |
| GB5Z | Renal tubulo-interstitial diseases, unspecified |  |
| GB60 | Acute kidney failure | Kidney failure |
| GB61 | Chronic kidney disease |  |
| GB6Z | Kidney failure, unspecified |  |
| GB70 | Calculus of upper urinary tract | Urolithiasis |
| GB71 | Calculus of lower urinary tract |  |
| GB7Z | Urolithiasis, unspecified |  |
| GB80 | Nonfamilial nongenetic cystic kidney disease | Cystic or dysplastic kidney disease |
| GB81 | Autosomal dominant polycystic kidney disease |  |
| GB82 | Autosomal dominant tubulointerstitial disease |  |
| GB83 | Nephronophthisis |  |
| GB8Y | Other specified cystic or dysplastic kidney disease |  |
| GB8Z | Cystic or dysplastic kidney disease, unspecified |  |
| GB90 | Certain specified disorders of kidney or ureter | Certain specified disorders of kidney or ureter |

**Supplementary Table 2:** **Comparison with previous work**. We show a list of datasets used in the previous work (Stanzick et al. 2021) and the data integrated in the current version of KidneyGPS (KidneyGPS 2.3).

| **Data** | **Stanzick et al. 2021** | **KidneyGPS 2.3** |
| --- | --- | --- |
| Credible set variants for independent eGFRcrea signals | Yes for 634 signals | Yes for 594 confirmed signals |
| Locus based validation with eGFRcys and BUN | Yes | Yes |
| Overlap with eGFRcrea decline associations | No | Yes |
| Differences of eGFRcrea associations in diabetes mellitus | No | Yes |
| Annotation of credible set variants regarding potential protein-relevant consequences | Yes, all variants restricted to CADD Phred Score ≥ 15 | Yes, only variants of category 3 (intron, upstream, downstream, 5’ UTR, 3’ UTR) restricted to CADD Phred Score ≥ 15 |
| Annotation of credible set variants regarding their impact on gene expression |  |  |
| - GTEx V7 kidney cortex | Yes | Yes |
| - NEPTUNE (glomerulus and tubulo-interstitium) | Yes | Yes |
| - Suzstaklab (glomerulus and tubulo-interstitium; Sheng et al. 2021) | No | Yes |
| - GTEx V7 other tissues | Yes | Yes |
| Annotation of credible set variants regarding their impact on gene splicing (GTEx V7) | Yes | Yes |
| Co-localization analyses of expression signals in kidney tissue (NEPTUNE) with association signals | Yes | Yes |
| Kidney Phenotypes in mice (MGI) | Yes | Yes |
| Genetic diseases with kidney phenotype in human |  |  |
| - OMIM | Yes | Yes |
| - Groopman et al. | Yes | Yes |
| - Wopperer et al. | No | Yes |
| Drug Information | No | Yes |

# **SUPPLEMENTARY FIGURES**

**Supplementary Figure 1:** **Comparison of the identifying p-values of the 634 signal index variants in analyses with different numbers of digits analysis**.

GWAS meta-analysis results for eGFRcrea in EUR with different numbers of decimal digits for effect estimates and standard errors (6/7 and 8/8) were conditioned with GCTA using the same 634 signal index variants and the 20K reference panel from UK Biobank as done in the original analysis by Stanzick et al [11]. P-values are shown on a -log10 scale. A: Comparison between the original analysis with 4/4 decimal digits with the re-analysis with 6/7 decimal digits. Shown are the identifying p-values of all 634 signal index variants. B: Zoom of blue box in panel A. Dots marked with the blue oval are index variants of a first signal in loci that are only significant in the all-ancestries analysis but not in EUR alone. C: Comparison of the identifying p-values of the 634 signal index variants between the re-analysis with 6/7 decimal digits and the re-analysis with 8/8 decimal digits.


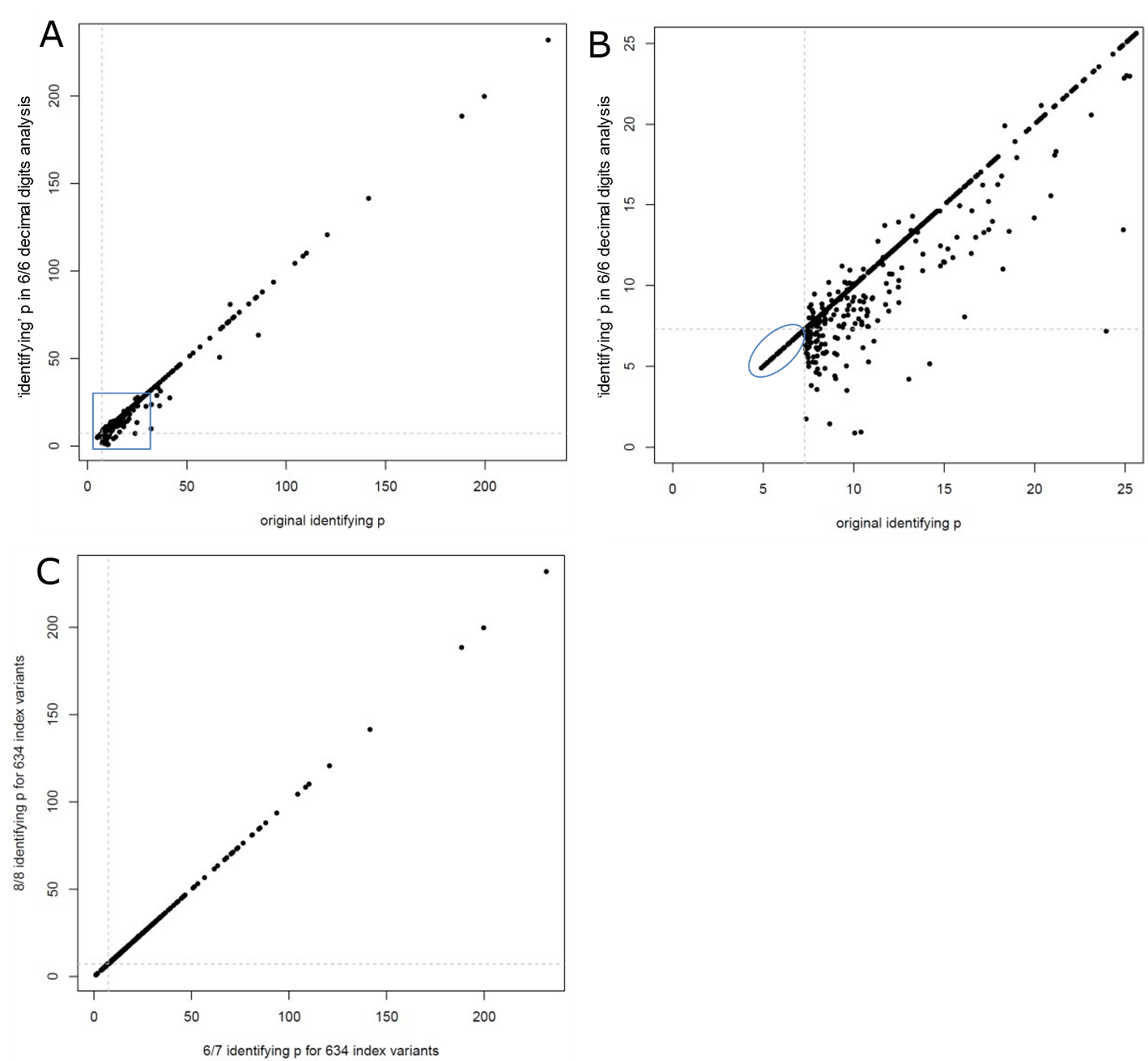


**Supplementary Figure 2:** **Comparison of credible set sizes for all 634 signals.** Based on fully conditioned results of the 6/7 and 8/8 decimal digits re-analyses, a PPA for each variant in each signal was calculated. 99% credible variant sets for each of the 634 signals were obtained by adding the variants with the highest PPA to the set until a cumulative PPA >99% was reached. A: Comparison of the credible set sizes of the original 4/4 decimal digits analysis with the 6/7 decimal digits analysis. B: Comparison of the credible set sizes of the 6/7 decimal digits analysis with the 8/8 decimal digits analysis. Small panels are focused on signals with 25 or less credible set variants.


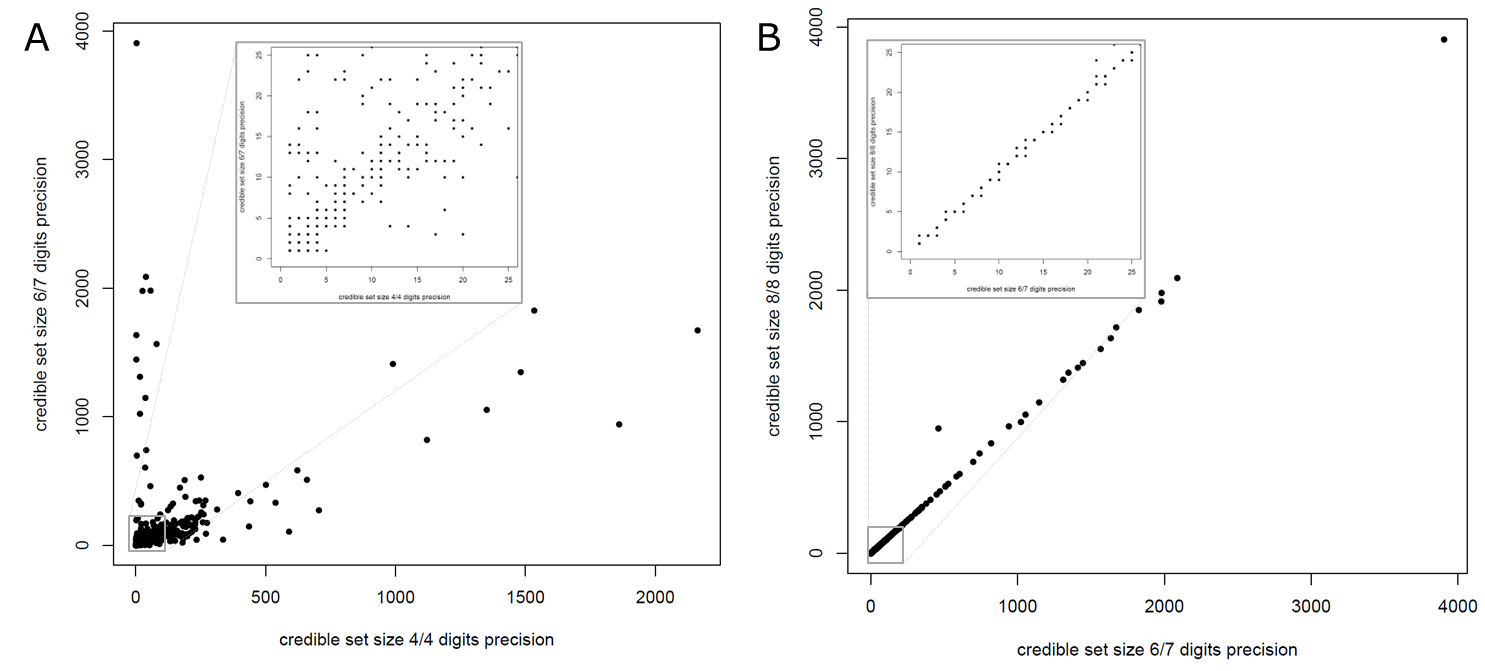


**Supplementary Figure 3.** **Comparison of variant’s PPA and 99% credible set size.**

For each of the 35,885 variants in the 594 99%-credible sets, we compared the variant’s PPA with the size of the credible set that contains the variant. Colour distinguishes small, medium, large and very large credible sets. Variants with higher PPA (>50%) cluster in signals with small credible set size. The plot shows variants in signals with maximum credible set size of 110 (79 signals and 22,040 variants in large credible sets omitted).


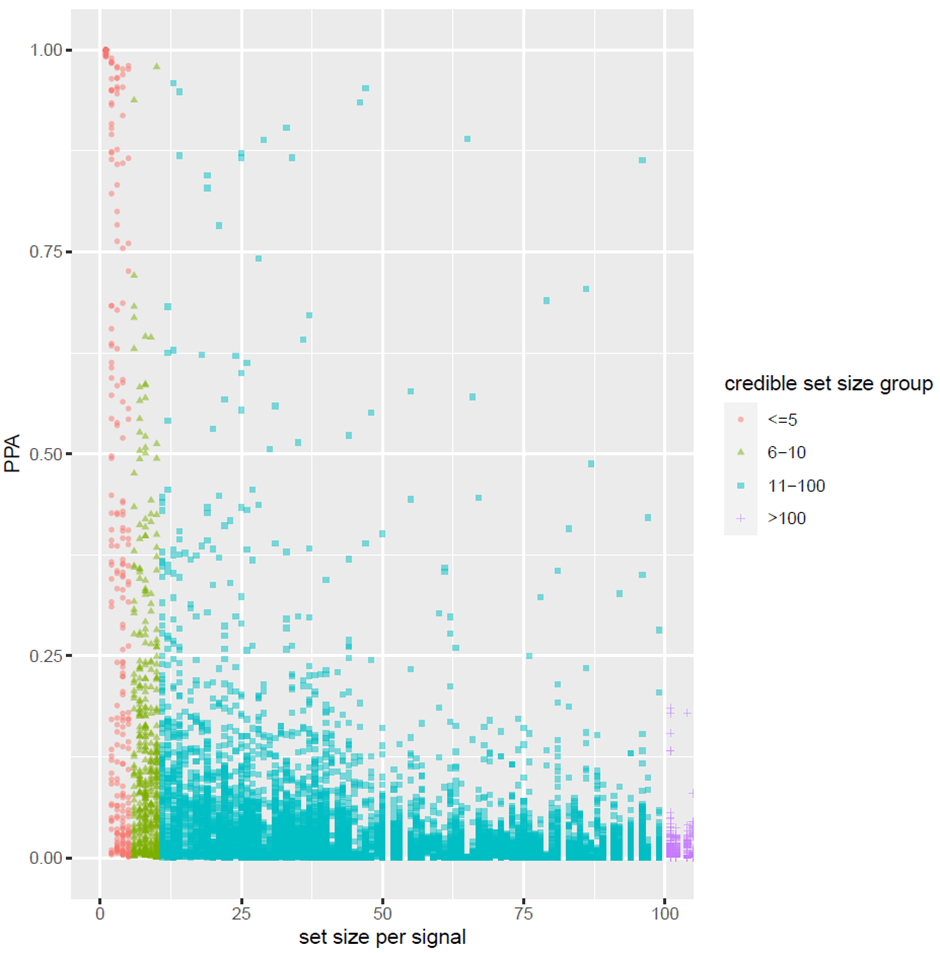


**Supplementary Figure 4: Architecture of KidneyGPS search functions.** This figure illustrates the three search options of KidneyGPS: Variant-search (by rs-identifier), Gene-search (by HGNC gene name), and Region-search (by chromosome and base-position). The user input is used directly (rs‑identifier, gene name) or indirectly (region leads to loci/signals and genes; gene leads to loci/signals) as a key to the underlying data in the KidneyGPS query. While the Variant- and Gene-search also provide the results of the individual queries as output, the result of the region search is the summary of the overlapping loci/signals and their properties, as well as the GPS table for the individual genes, which is a summary of the Gene-search and also appears as output with it.


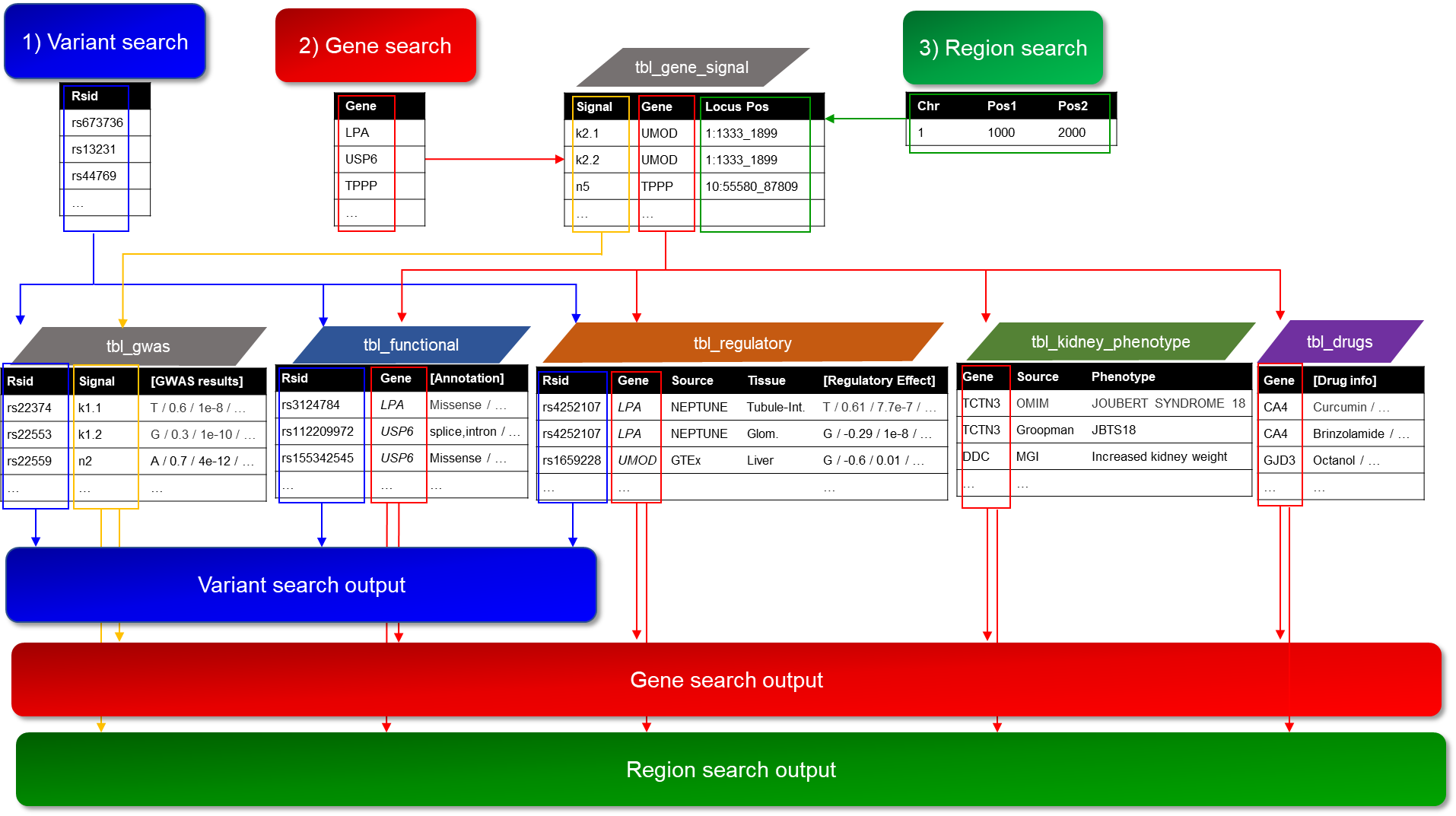
**^[[1]](#footnote-1)^**

References

1. Sollis E, Mosaku A, Abid A, Buniello A, Cerezo M, Gil L, et al. The NHGRI-EBI GWAS Catalog: knowledgebase and deposition resource. Nucleic Acids Research. 2023;51:D977-D985. doi:10.1093/nar/gkac1010.

2. Ghoussaini M, Mountjoy E, Carmona M, Peat G, Schmidt EM, Hercules A, et al. Open Targets Genetics: systematic identification of trait-associated genes using large-scale genetics and functional genomics. Nucleic Acids Research. 2021;49:D1311-D1320. doi:10.1093/nar/gkaa840.

3. Common Metabolic Diseases Knowledge Portal (cmdkp.org). https://hugeamp.org/dinspector.html?dataset=Stanzick2021_CKD_mixed&phenotype=eGFRcrea. Accessed 16 Feb 2023.

4. Liu H, Doke T, Guo D, Sheng X, Ma Z, Park J, et al. Epigenomic and transcriptomic analyses define core cell types, genes and targetable mechanisms for kidney disease. Nature Genetics. 2022;54:950–62. doi:10.1038/s41588-022-01097-w.

5. Ochoa D, Hercules A, Carmona M, Suveges D, Baker J, Malangone C, et al. The next-generation Open Targets Platform: reimagined, redesigned, rebuilt. Nucleic Acids Research. 2023;51:D1353-D1359. doi:10.1093/nar/gkac1046.

6. Hamosh A, Scott AF, Amberger J, Valle D, McKusick VA. Online Mendelian Inheritance In Man (OMIM). Human Mutation. 2000;15:57–61. doi:10.1002/(SICI)1098-1004(200001)15:1<57::AID-HUMU12>3.0.CO;2-G.

7. Groza T, Gomez FL, Mashhadi HH, Muñoz-Fuentes V, Gunes O, Wilson R, et al. The International Mouse Phenotyping Consortium: comprehensive knockout phenotyping underpinning the study of human disease. Nucleic Acids Research. 2023;51:D1038-D1045. doi:10.1093/nar/gkac972.

8. Karczewski KJ, Francioli LC, Tiao G, Cummings BB, Alföldi J, Wang Q, et al. The mutational constraint spectrum quantified from variation in 141,456 humans. Nature. 2020;581:434–43. doi:10.1038/s41586-020-2308-7.

9. Stelzer G, Rosen N, Plaschkes I, Zimmerman S, Twik M, Fishilevich S, et al. The GeneCards Suite: From Gene Data Mining to Disease Genome Sequence Analyses. Current Protocols in Bioinformatics. 2016;54:1.30.1-1.30.33. doi:10.1002/cpbi.5.

10. Pattaro C, Teumer A, Gorski M, Chu AY, Li M, Mijatovic V, et al. Genetic associations at 53 loci highlight cell types and biological pathways relevant for kidney function. Nat Commun. 2016;7:10023. doi:10.1038/ncomms10023.

11. Stanzick KJ, Li Y, Schlosser P, Gorski M, Wuttke M, Thomas LF, et al. Discovery and prioritization of variants and genes for kidney function in 1.2 million individuals. Nat Commun. 2021;12:4350. doi:10.1038/s41467-021-24491-0.

1. Following columns are included in

   [GWAS results]: Chr, Pos, Effect allele, Other allele, N, EAF, Association with log(eGFR) unconditioned all ancestries (beta, StdErr, P-value), Association with log(eGFR) unconditioned European ancestry (beta, StdErr, P-value), Association with log(eGFR) conditioned on other signal index variants (beta, StdErr, P-value), Nearest gene, Locus ID, Signal ID, PPA (if applicable)

   [Annotation]: PPA, Locus ID, Signal ID, Reference allele, Alternative allele, Aminoacid position, Reference aminoacid,CADD Phred-Score

   [Regulatory Effect]: PPA, Effect allele, Other allele, EAF, Effect direction on expression, Effect on expression, StdErr, P-value

   [Drug info]: All respective target genes, Target name, Targettype, Highest drug status, Mode of action, Disease/Indication [↑](#footnote-ref-1)
